# The Novel RXR Agonist MSU-42011 Differentially Regulates Gene Expression in Mammary Tumors of MMTV-Neu Mice

**DOI:** 10.3390/ijms24054298

**Published:** 2023-02-21

**Authors:** Lyndsey A. Reich, Ana S. Leal, Edmund Ellsworth, Karen T. Liby

**Affiliations:** 1 Department of Pharmacology and Toxicology, College of Osteopathic Medicine, East Lansing, MI 48824, USA; 2 Medicinal Chemistry Facility, Michigan State University, East Lansing, MI 48824, USA; 3 Department of Pharmacology and Toxicology, Michigan State University, B430 Life Science Building, 1355 Bogue Street, East Lansing, MI 48824, USA

**Keywords:** RXR agonist, breast cancer, transcriptomics

## Abstract

Retinoid X receptor (RXR) agonists, which activate the RXR nuclear receptor, are effective in multiple preclinical cancer models for both treatment and prevention. While RXR is the direct target of these compounds, the downstream changes in gene expression differ between compounds. RNA sequencing was used to elucidate the effects of the novel RXRα agonist MSU-42011 on the transcriptome in mammary tumors of HER2+ mouse mammary tumor virus (MMTV)-Neu mice. For comparison, mammary tumors treated with the FDA approved RXR agonist bexarotene were also analyzed. Each treatment differentially regulated cancer-relevant gene categories, including focal adhesion, extracellular matrix, and immune pathways. The most prominent genes altered by RXR agonists positively correlate with survival in breast cancer patients. While MSU-42011 and bexarotene act on many common pathways, these experiments highlight the differences in gene expression between these two RXR agonists. MSU-42011 targets immune regulatory and biosynthetic pathways, while bexarotene acts on several proteoglycan and matrix metalloproteinase pathways. Exploration of these differential effects on gene transcription may lead to an increased understanding of the complex biology behind RXR agonists and how the activities of this diverse class of compounds can be utilized to treat cancer.

## 1. Introduction

Retinoid X receptor (RXR) agonists bind to and activate the nuclear receptor RXR. RXR is a type II nuclear receptor, which is found in the nucleus bound to DNA and corepressor proteins [1,2]. Upon activation by a ligand, conformational changes in the structure of RXR promote dissociation of corepressor proteins and recruitment of diverse coactivator proteins. Because of its flexible dimerization domain, RXR homodimerizes or heterodimerizes with other nuclear receptors, including peroxisome proliferator-activated receptor (PPAR), liver X receptor (LXR), pregnane X receptor (PXR), or vitamin D receptor (VDR), to initiate transcription [3]. Upon activation, RXR regulates the transcription of target genes, involved in proliferation, differentiation, survival, and immune cell function [4].

Bexarotene is an RXR agonist, currently FDA approved to treat cutaneous T cell lymphoma (CTCL) [5]. Bexarotene has been tested in clinical trials for breast and non-small cell lung cancer but failed to attain approval for these indications, despite promising responses in some patients and manageable side effects [6,7]. Many have sought to improve the efficacy of bexarotene via novel drug delivery systems and formulations [8] or have made structural modifications to identify new RXR agonists [9,10]. Our new analog, MSU-42011, is effective for treatment in the MMTV-Neu model of HER2+ breast cancer [11], an established mouse model which recapitulates the human disease, as has been validated by gene expression profiling [12,13]. This model expresses wild-type, unactivated Neu in mammary tissue under the mouse mammary tumor virus (MMTV) promoter [14]. MSU-42011 also effectively reduces established tumor burden in the A/J mouse model of carcinogen-induced lung cancer [9]. In both of these preclinical models, changes in immune cell populations differed in the tumors of mice treated with MSU-42011 vs. bexarotene [9], suggesting that these compounds have distinct patterns of immunomodulatory activity.

Nuclear receptor biology is complex, and gene transcription varies based on the nuclear receptor binding partner of RXR [15]. For example, target pathways under the control of RXR:RAR heterodimers include genes which induce the enzymes phosphoenolpyruvate carboxykinase (PEPCK) and tissue transglutaminase 2 (TG2), immune-related genes such as B cell translocation gene 2 (*Btg2*), and retinoic acid response genes such as aberrant cellular retinol binding protein 1 (*Crbp1*) and cellular retinoic acid-binding protein 1 (*Crabp1*) [16]. Several genes involved in lipogenesis (*Agpat2*, *Acsl1*, *Gpat3*) and glucose metabolism (*Hk2*, *Taldo1*) are regulated by RXR:PPAR dimerization in adipocytes [17]. VDR, another nuclear receptor for which RXR is an obligate heterodimer, regulates expression of an extensive list of genes which act as VDR response elements. In quiescent hepatic stellate cells, binding of calcipotriol to the VDR nuclear receptor initiates binding to a cistrome of 6281 target sites, which expands to 24,984 sites when these cells are activated by lipopolysaccharide (LPS) or transforming growth factor beta (TGFβ) [18]. Through dimerization with the PXR nuclear receptor, RXR regulates transcription of genes involved in xenobiotic and endobiotic metabolism, cytoprotective mechanisms, and detoxification, including enzymes such as CYP3A4 and efflux pumps such as MDR1 [19,20]. Because the network of nuclear receptor target genes is vast, the biological effects of RXR activation are numerous and diverse.

Others have previously investigated the effects of bexarotene on the transcriptional regulatory network in mammary glands of mouse models of breast cancer [21], but to date no one has analyzed gene expression data from tumors treated with different RXR agonists. To this end, we used RNA sequencing to compare pathways activated by treatment with MSU-42011 versus pathways activated by bexarotene and validated selected genes by qPCR and immunohistochemistry. These data provide additional information about the cancer-relevant transcriptional regulation of RXR agonists and the diversity of activities of these compounds.

## 2. Results

### 2.1. RXR Agonists Regulate Pathways Relevant in Breast Cancer

**Figure 1:**
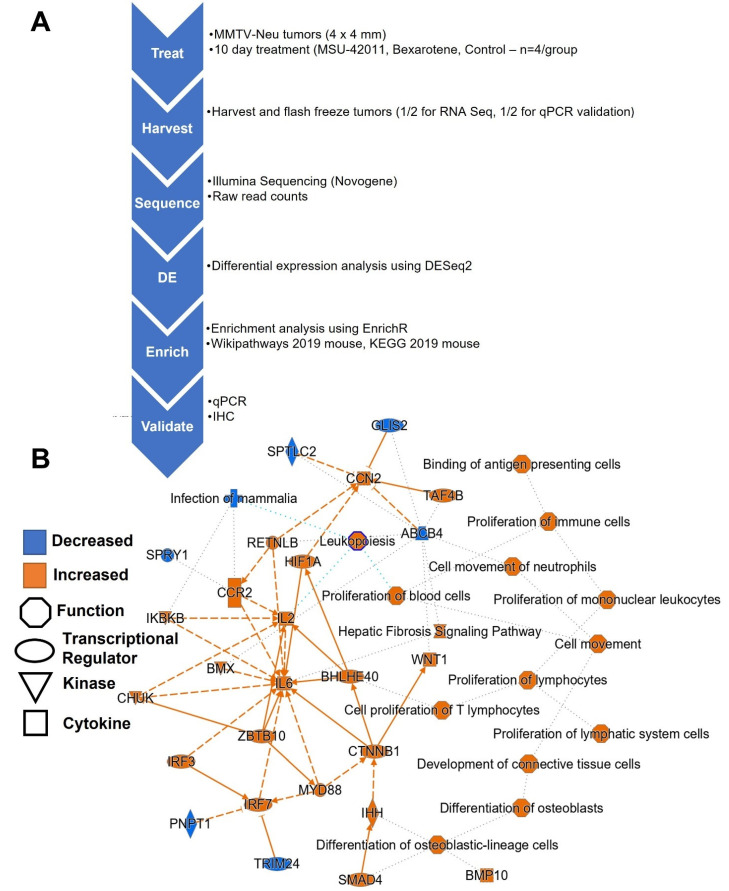
RXR agonists regulate pathways relevant in breast cancer. (**A**) Experimental flow diagram of RNA Sequencing studies. (**B**) Qiagen Ingenuity Pathway Analysis reveals a network of pathways differentially regulated by the RXR agonists bexarotene and MSU-42011.

To characterize differential expression across the whole transcriptome, high-throughput techniques such as RNA sequencing (RNA-seq) allow us to parse differentially expressed genes into biological pathways for comprehensive analysis of RXR agonist response in tumors. For these studies, MMTV-neu mice (four per group) were fed control diet, MSU-42011 (100 mg/kg diet), or bexarotene (100 mg/kg diet) for 10 days. Tumors were harvested and RNA was analyzed by RNA-seq (Figure 1A). Relative to control tumors, tumors treated with both RXR agonists had higher expression of canonical immune pathways such as binding of antigen presenting cells and proliferation of immune cells, mononuclear leukocytes, and lymphocytes (Figure 1B). Causal network analysis [22], a means of identifying upstream regulators of differentially expressed genes from RNA-seq, identified SMAD4, IRF3, IRF7, and ZBTB10 as possible regulatory nodes.

### 2.2. Top Genes Differentially Expressed in Tumors Treated with MSU-42011 and Bexarotene Correlate with Patient Survival

**Figure 2:**
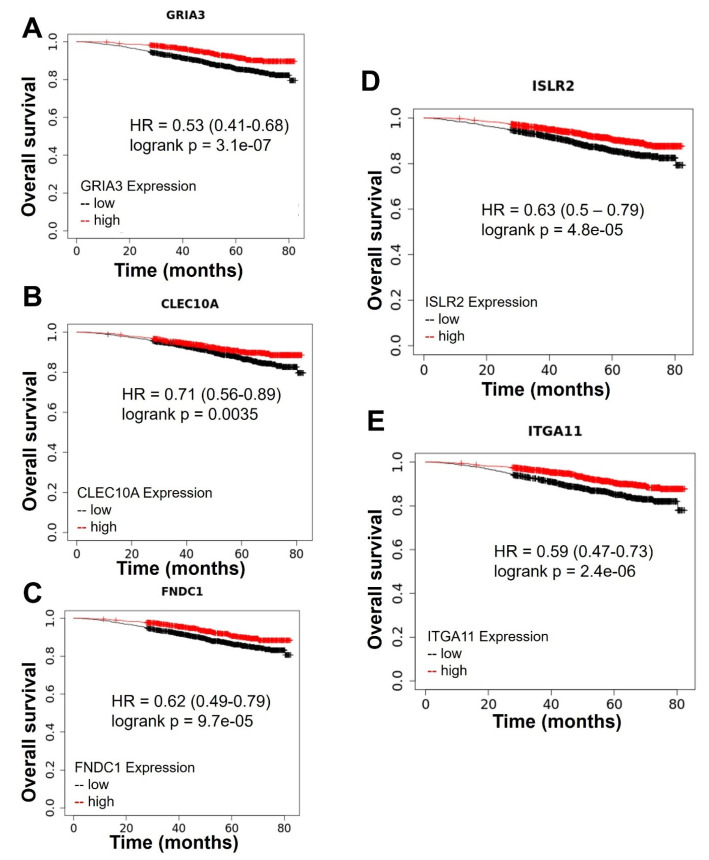
Top genes differentially expressed in tumors treated with MSU-42011 and bexarotene correlate with patient survival. Kaplan–Meier survival curves from the top 10 differentially expressed genes from MSU-42011 vs. bexarotene vs. control where expression is correlated with overall survival in breast cancer patients [n = 2976; 1259 high 1717 low in (**A**), 1220 high 1756 low in (**B**), 1252 high 1724 low in (**C**), 1588 high 1388 low in (**D**), 1703 high 1273 low in (**E**)]. Data generated using KMPlot (http://www.kmplot.com, accessed on 26 July 2022). No stratification strategies were used. Cutoffs for high and low expression were determined by auto-cutoff tool based on false discovery rate and *p* value 1.

Differential expression analysis revealed a list of genes (GSE211290) differentially expressed in control tumors vs. tumors from mice treated with MSU-42011 vs. tumors from mice treated with bexarotene. This list of 289 significantly (padj < 0.05) upregulated or significantly downregulated genes was sorted by adjusted *p* value. Of the top 10 most significant differentially expressed genes, high levels of expression of five genes correlate with improved overall survival in breast cancer patients—*GRIA3* (logrank *p* = 3.1 × 10^−7^) (Figure 2A), *CLEC10* (logrank *p* = 0.0035) (Figure 2B), *FNDC1* (logrank *p* = 9.7 × 10^−5^) (Figure 2C), *ISLR2* (logrank *p* = 4.8 × 10^−5^) (Figure 2D), and *ITGA11* (logrank *p* = 2.4 × 10^−6^) [23] (Figure 2E). Survival curves were generated using the Kaplan–Meier Plotter (KmPlot) [24], without further stratification of breast cancer patients. These genes code for a glutamate receptor linked to migration and invasion (*GRIA3*) [25]; a c-type lectin with a role in cellular adhesion, signaling, and inflammation which serves as a dendritic cell marker (*CLEC10*) [26]; a fibronectin protein associated with invasion and chemoresistance (*FNDC1*) [27]; a member of the immunoglobulin superfamily which participates in nervous system development (*ISLR2*) [28]; and an alpha integrin which regulates adhesion to the extracellular matrix and the organization of collagen (*ITGA11*) [29].

### 2.3. RXR Agonists Regulate Cancer-Relevant Biological Pathways in MMTV-Neu Tumors

**Figure 3:**
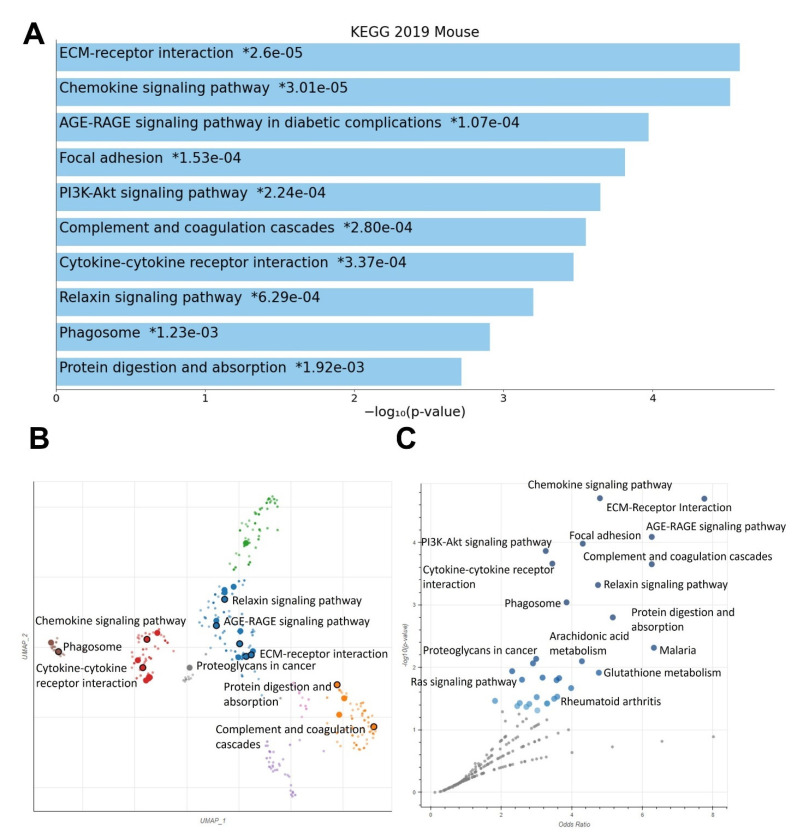
RXR agonists regulate cancer-relevant biological pathways in MMTV-Neu tumors. Female MMTV-Neu mice with established mammary tumors 4 mm in diameter were fed control diet, bexarotene (100 mg/kg in diet), or MSU-42011 (100 mg/kg in diet) for 10 days. KEGG 2019 mouse was used as the database for enrichment analysis of both RXR agonists. (**A**) Bar graph of top differentially expressed pathways. Scatterplot (**B**) and volcano plot (**C**) of top differentially expressed pathways. * = *p*-value.

Enrichment analysis on control vs. MSU-42011 vs. bexarotene differential expression data using EnrichR reveals a set of pathways regulated by treatment with the various RXR agonists (Figure 3). The KEGG 2019 mouse database was used for these analyses; analysis using the Wikipathways 2019 mouse database is also shown (Supplemental Figure S1). Identified pathways include genes associated with ECM-receptor interaction, chemokine signaling, focal adhesion, PI3K-Akt signaling, complement and coagulation cascades, and the phagosome. Genes within these pathways encode for macromolecules involved in cellular structure and function, cellular behavior such as adhesion and migration, and downstream signaling pathways.

### 2.4. MSU-42011 and Bexarotene Induce Unique Gene Expression Profiles with Some Unifying Characteristics in Treated Tumors of a HER2+ Murine Model

**Figure 4:**
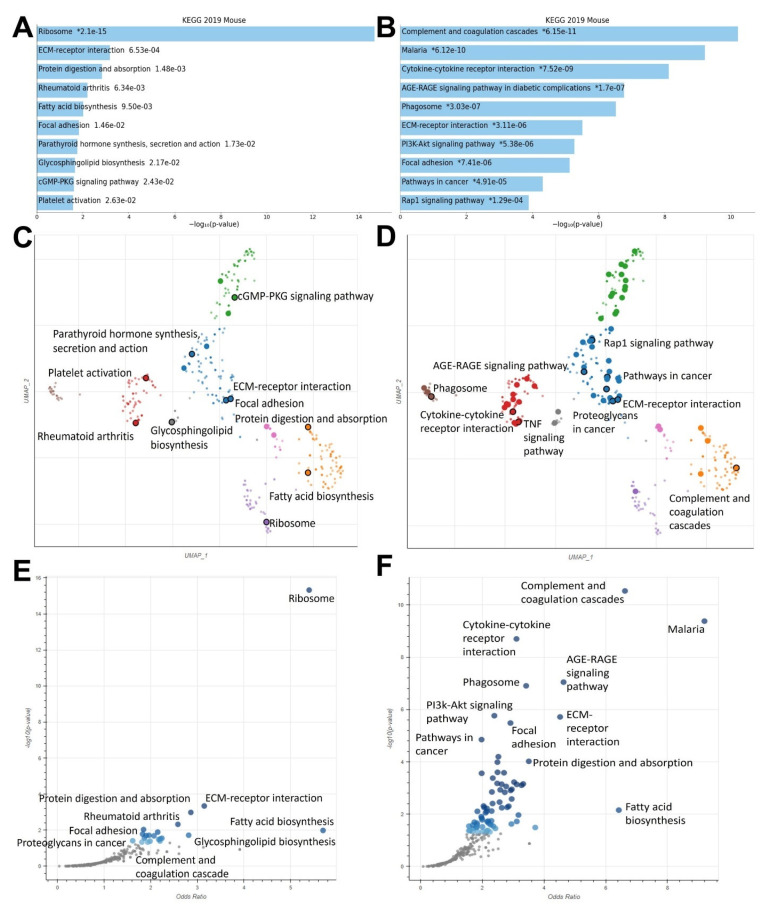
MSU-42011 and bexarotene induce unique gene expression profiles with some unifying characteristics in treated mammary tumors of a HER2+ murine model. (**A**,**B**) Bar graph of differentially regulated pathways in MSU 42011-treated vs. control tumors (**A**) and bexarotene-treated vs. control tumors (**B**) using the KEGG 2019 Mouse database in Enrichr. (**C**–**F**) Scatterplot (**C**,**D**) and volcano plot (**E**,**F**) depictions of differentially regulated pathways in MSU-42011-treated vs. control tumors (**C**,**E**) and bexarotene-treated vs. control tumors (**D**,**F**). * = *p*-value.

Enrichment analysis was used to compare differentially expressed genes in control vs. MSU-42011 and control vs. bexarotene groups. Bar charts of these analyses reveal enrichment of shared pathways (focal adhesion, ECM-receptor interaction), as well as pathways unique to MSU-42011 (rheumatoid arthritis, ribosome) and pathways unique to bexarotene (PI3K-Akt signaling pathway, Rap1 signaling pathway) (Figure 4A,B). These unique pathways include genes which code for critical components related to cellular proliferation, immunity, and cellular migration and invasion. Scatterplot depictions of pathways regulated by MSU-42011 (Figure 4C) and by bexarotene (Figure 4D) highlight the similarities and differences in pathway enrichment within a particular cluster across different drug treatments. Volcano plot depictions of pathways regulated by MSU-42011 (Figure 4E) and bexarotene (Figure 4F) highlight the pathways unique to MSU-42011, especially the ribosome pathway. This pathway contains genes which encode for components necessary for rapid cellular turnover, which is particularly relevant to tumor biology [30,31]. KEGG 2019 was used as a database for these analyses.

### 2.5. MSU-42011 Increases Col6a3 and Map9 Expression in Mouse Mammary Tumors

**Figure 5:**
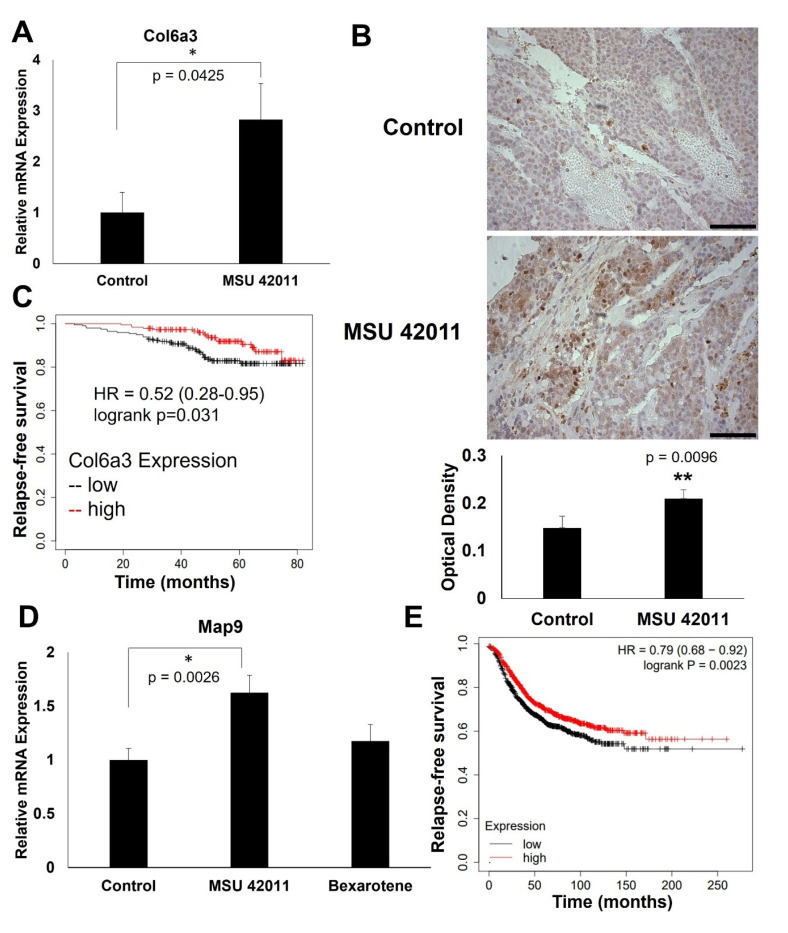
MSU-42011 increases *Col6a3* and *Map9* expression in mouse mammary tumors. Tumors treated with MSU-42011 (100 mg/kg diet) or control were harvested and flash-frozen. Tumors were homogenized and RNA extracted. (**A**,**D**) mRNA expression was detected by qPCR. N = 5 mice/group. Error bars represent standard error. * *p* < 0.05; ** *p* < 0.01 (**B**). Immunohistochemical staining for Col6a3, representative of four mice per group and quantified using ImageJ. Scale bar = 60 μm, 40× magnification (**C**,**E**) Kmplot survival curves correlate expression of Col6a3 (**C**) with survival in HER2+ breast cancer patients (n = 379; 183 high, 196 low) and Map9 (**E**) with survival in breast cancer patients (n = 2032; 1015 high, 1017 low).

Several genes were selected from the differential expression analysis for validation of mRNA expression by qPCR and protein levels by IHC. Collagen type VI a3 chain (COL6A3) is an extracellular matrix protein which is altered in several types of cancer [32]. *Col6a3* mRNA expression (Figure 5A) is increased in tumors treated with MSU-42011 (*p* = 0.0425) but not in tumors treated with bexarotene. IHC (Figure 5B) demonstrates a 41% increase in Col6a3 protein levels in tumors treated with MSU-42011 (*p* = 0.0096), and no apparent increase in Col6a3 in bexarotene-treated tumors (Supplemental Figure S2D). Kmplot was used to investigate the relevance of *Col6a3* expression in human breast tumors (Figure 5C). High expression of *COL6A3* is correlated with increased relapse-free survival (*p* = 0.031) in HER2+ breast cancer patients. qPCR (Figure 5D) also confirms a significant (*p* = 0.0026) increase in *Map9* mRNA in MSU-42011-treated tumors, while there was no significant increase observed in bexarotene-treated tumors. MAP9 is a microtubule-associated protein which regulates cell cycle and the DNA damage response [33]. High expression of *MAP9* is positively correlated with relapse-free survival (Figure 5E) in breast cancer patients (*p* = 0.0023).

### 2.6. MSU-42011 Increases IL-18 and H2-AA Expression in Mouse Mammary Tumors

**Figure 6:**
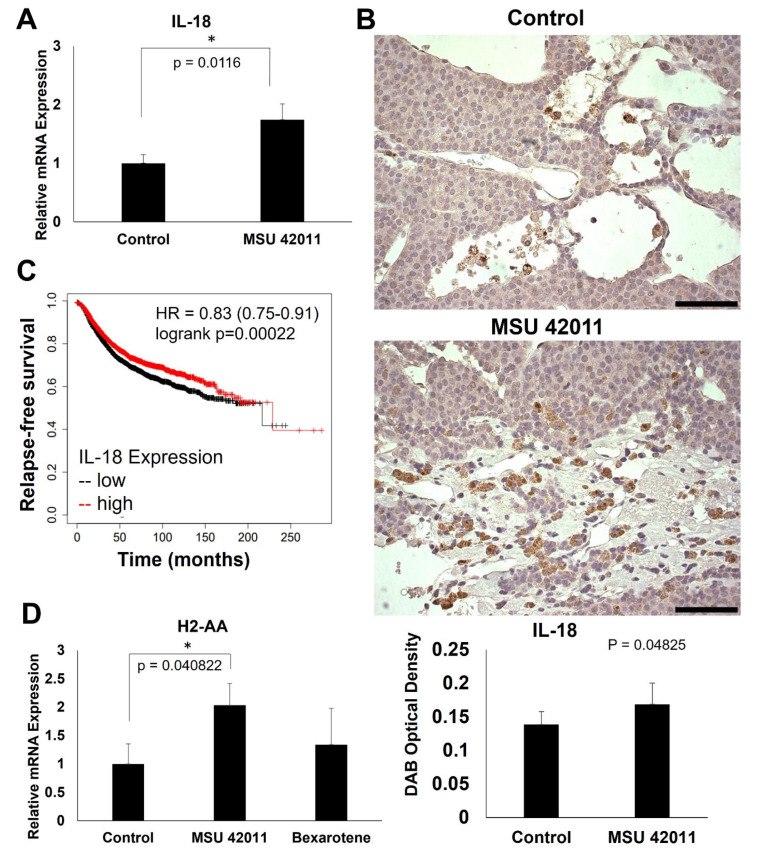
MSU-42011 increases IL-18 and H2-AA expression in mouse mammary tumors. Tumors treated with MSU-42011 (100 mg/kg diet) or control were harvested and flash-frozen. Tumors were homogenized and RNA extracted. (**A**,**D**) Gene expression was detected by qPCR. **N** = 5 mice/group. Error bars represent standard error. * *p* < 0.05 (**B**). Immunohistochemical staining for IL-18, representative of four mice per group and quantified using ImageJ. Scale bar represents 60 μm, 40× magnification (**C**) Kmplot survival curve correlates expression of Il-18 with survival in breast cancer patients (n = 4929; 2456 high, 2473 low).

As shown in Figure 4, the rheumatoid arthritis pathway is differentially regulated by MSU-42011 but not by bexarotene. The genes within this pathway include immune response genes which may contribute to the anti-tumor immunomodulatory activity of MSU-42011 [34]. The cytokine *IL-18* was selected from the rheumatoid arthritis pathway for validation (Figure 6A). In tumors of mice treated with MSU-42011, but not bexarotene (Supplemental Figure S2A), mRNA expression of *IL-18* increased (*p* = 0.0116). IHC (Figure 6B) revealed an increase in IL-18 in tumors treated with MSU-42011 (*p* = 0.04825).

Interestingly, tumors from the bexarotene group display an apparent paucity of IL-18, even in comparison to control tumors (Supplemental Figure S2B). Importantly, Kmplot analysis reveals that high IL-18 expression is correlated with increased relapse-free survival in breast cancer patients (*p* = 0.00022) (Figure 6C). MSU-42011-treated tumors also demonstrate a significant (*p* = 0.040822) upregulation of the gene coding for major histocompatibility complex (MHC) component H2-AA by qPCR (Figure 6D).

### 2.7. MSU-42011 Polarizes Bone Marrow-Derived Macrophages (BMDMs) towards an Anti-Tumor Phenotype

**Figure 7:**
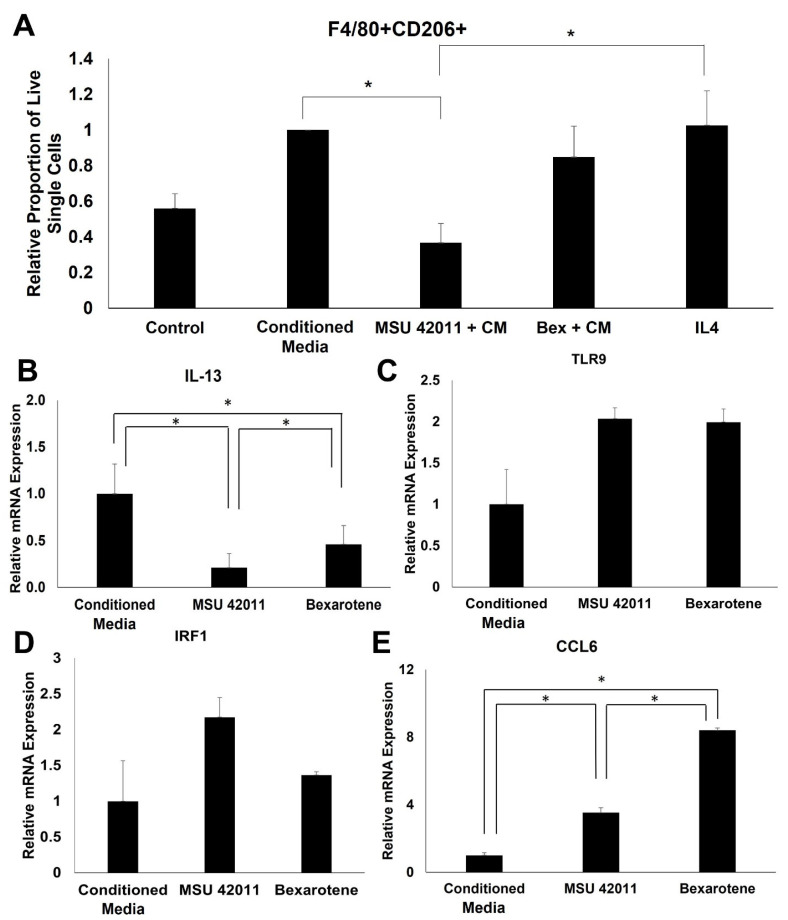
MSU-42011 polarizes bone marrow-derived macrophages (BMDMs) towards an anti-tumor phenotype. BMDMs were isolated from C57BL/6 mice and differentiated with MCSF. On day 5, BMDMs were treated with conditioned media (CM) from E18-14C-27 cells, either alone or with 300 nM RXR agonist. (**A**) BMDMs were harvested for flow cytometry to detect percentage of live single cells that were F4/80 + CD206+. All data normalized to conditioned media-treated cells and presented as mean of three experimental runs. Error bars represent standard error. (**B**–**E**) RNA was extracted, and gene expression was detected by qPCR. Error bars represent standard error of three biological replicates. * *p* < 0.05.

RXR agonists regulate pathways relevant to the function of the immune system, such as rheumatoid arthritis, complement and coagulation cascade, and cytokine–cytokine receptor interaction. To validate and further characterize the immunomodulatory activity of these compounds, BMDMs treated with RXR agonists were evaluated for expression of cancer-relevant genes within these pathways. Monocytes were harvested and differentiated with MCSF (20 ng/mL). On Day 5, BMDMs were treated with conditioned media from E18-14C-27 cells, derived from MMTV-Neu mammary tumors, to induce a tumor-educated macrophage phenotype. BMDMs were treated with conditioned media alone, or with 300 nM of either MSU-42011 or bexarotene. After 24 h, the relative proportion of F4/80+CD206+ macrophages was significantly (*p* = 0.02726) lower in BMDMs treated with conditioned media and 300 nM MSU-42011 compared to conditioned media alone (Figure 7A) In comparison, treatment with 300 nM bexarotene and conditioned media did not significantly alter the relative proportion of F4/80 + CD206+ BMDMs (*p* = 0.9423). Treatment with 300 nM of either RXR agonist significantly (*p* = 0.0016) decreased mRNA expression of IL-13, an immunosuppressive cytokine (Figure 7B). A trend of increasing *TLR9* and *IRF1* mRNA expression, associated with a pro-inflammatory, anti-tumor phenotype was observed in BMDMs treated with both RXR agonists (Figure 7C,D). RXR agonists also induce a significant (*p* = 0.00015) increase in expression of CCL6, a pro-inflammatory cytokine (Figure 7E).

## 3. Discussion

RXR agonists are a class of drugs with anti-tumor activity in preclinical models of breast and lung cancer [9,10,35]. While the known target of these drugs is the nuclear receptor RXR, different RXR agonists have markedly different effects on downstream gene expression. The nature of nuclear receptors—their ability to homodimerize or to heterodimerize with other nuclear receptors, the diversity of the structures of their ligands, and the vast number of target genes—makes RXR an interesting drug target. These characteristics likely differ among RXR agonists, potentially initiating heterodimerization with different nuclear receptor partners or recruiting different coactivators, leading to variations in resulting gene expression which may be clinically beneficial.

For the first time, using RNA-seq, we compared pathway activation and biological activity of the novel RXR agonist MSU-42011 and the FDA-approved bexarotene. The regulation of many similar pathways, including focal adhesion and extracellular matrix components, are shared by these two molecules (Figure 4). Immune-related pathways such as cytokine signaling pathways, complement activation, and genes related to phagosome activity are also shared by both MSU-42011 and bexarotene. Interestingly, validation of individual genes within these pathways shows that while one RXR agonist upregulates an immune- or ECM-related gene, the other RXR agonist does not. For example, MSU-42011 increases expression of *Il-18* and *Col6a3* at both the mRNA and protein level (Figure 5 and Figure 6), but neither of these two gene products are increased in tumors treated with bexarotene.

Several pathways were identified through enrichment analysis that were unique to a single RXR agonist. For example, the ribosome pathway and the fatty acid biosynthesis pathway, through which macromolecules critical to cellular function are synthesized, were prominent in enrichment analysis for MSU-42011 but not bexarotene. Conversely, the proteoglycans in cancer pathway, containing genes which code for matrix metalloproteinases (MMP), WNT signaling molecules, and growth factors such as IGF1 and FGF2, is prominent in bexarotene differential expression analysis but not MSU-42011.

The increase in *Il-18* expression seen at both the level of mRNA and protein in tumors treated with MSU-42011, but not bexarotene, suggests that this RXR agonist promotes a pro-inflammatory tumor microenvironment, which can be harnessed for breast cancer treatment. *IL-18* expression has been investigated as a possible prognostic indicator in breast cancer patients [36] and augments the cytotoxicity of NK cells [37]. Further investigation into the mechanism of MSU-42011 is necessary to determine if *Il-18* is a critical mediator of anti-tumor immune response, and if it can be used as an indicator of response to therapy.

Furthermore, the increase in *H2-Aa* mRNA observed in tumors treated with MSU-42011 provides further evidence of its immune modulatory properties. H2-AA is an MHC class II component, higher expression of which is correlated with increased survival in ovarian cancer [38]. MHC II is responsible for antigen presentation to CD4+ T cells, which have recently gained recognition supporting the activation of cytotoxic T cells and mediating checkpoint inhibition response in cancer [39]. The MHC II pathway is necessary for antitumor immunity in several cancer types and is upregulated by treatment with histone deacetylase (HDAC) inhibitors [40,41]. In triple negative breast cancer, high expression of genes associated with the MHC II pathway correlates with progression-free survival [42]. Pharmacologic means of augmenting MHC II signaling may be a valuable therapeutic strategy for enhancing anti-tumor immunity. The increase in expression in *Il-18* mRNA and protein and *H2-aa* mRNA observed in tumors treated with MSU-42011, but not bexarotene, may provide insight into the unique immunomodulatory properties of these two RXR agonists.

While *COL6A3* expression has been explored as a prognostic biomarker in colorectal cancer [43], less is known about the role of *COL6A3* in breast cancer. There is a trend of decreased *COL6A3* expression with increasing tumor stage in breast cancer patients [32], which suggests a propensity for invasion and metastasis in these tumors [44]. Further, increased expression of *COL6A3* in breast cancer after chemotherapy may predict for responsiveness to chemotherapy [45]. Finally, a cleavage fragment of COL6A3 known as endotrophin recruits macrophages through induction of monocyte chemoattractant protein-1 (MCP1) and increases IL-6 and TNFα in the tumor microenvironment [46]. Similarly, in obesity, collagen VI expression in omental white adipose tissue is correlated with expression of MCP-1, CD68, and CD86, providing further evidence that this collagen influences macrophage infiltration and phenotype [47]. As the role of COL6A3 is complex and can vary between cancer types and across tumor staging, the increase in expression of *Col6a3* mRNA and protein in tumors treated with MSU-42011 and resultant effect on invasion and immunity merits further investigation.

The expression of the microtubule-associated protein MAP9 is altered in both colorectal cancer and breast cancer, leading to cell cycle dysregulation [33]. *MAP9* hypermethylation in breast cancer leads to decreased expression and may have utility as an epigenetic biomarker [48]. Further, *MAP9* transcription is induced upon DNA damage, and MAP9 protein interacts with and stabilizes p53 in Sa-OS-2 cells, leading to increased tumor suppressor activity [49]. As mRNA expression of *Map9* is increased in tumors treated with MSU-42011, an exploration of the effects of MSU-42011 on cell cycle control and the ways this may be exploited for therapeutic purposes is warranted.

Based on our RNA sequencing data, particularly differentially expressed genes and pathways relating to immunity, we investigated the effects of MSU-42011 treatment on cell surface marker and gene expression in BMDMs (Figure 7). MSU-42011 decreased the relative proportion of F4/80 + CD206+ BMDMs by flow cytometry, indicating that treatment with MSU-42011 decreases immunosuppressive macrophages, while bexarotene did not have any effect. Further markers of immunosuppressive and pro-inflammatory macrophages were evaluated in BMDMs treated with RXR agonists by qPCR. MSU-42011 decreased expression of *Il-13*, an immunosuppressive cytokine, and increased expression of *Ccl6*, a pro-inflammatory cytokine. Furthermore, treatment with MSU-42011 increased expression of *Tlr9* and *Irf1*, an interferon-regulatory factor known to be induced by ligation of TLR9. The TLR9-IRF1-IFN signaling axis has been implicated in macrophage polarization [50]. Taken together, these data provide additional evidence that MSU-42011 skews macrophages away from a tumor-promoting, immunosuppressive phenotype and toward an anti-tumor, proinflammatory phenotype. This effect on macrophages may be important for the anti-tumor activity of MSU-42011.

In conclusion, treatment with RXR agonists results in modulation of gene expression that are consistent with effective cancer treatments. As a drug class, RXR agonists display a broad range of activities, regulating different genes and biological pathways. The diversity of these compounds may allow them to be utilized for targeted or personalized cancer therapy.

## 4. Materials and Methods

### 4.1. Drugs

MSU-42011 was prepared as previously described [9,10,11]. Bexarotene was purchased from LC Laboratories (Woburn, MA, USA). For in vivo studies, RXR agonists were dissolved in a vehicle of 1 part ethanol: 3 parts highly purified coconut oil (Neobee oil, Thermo Fisher Scientific, Waltham, MA, USA). A total of 50 mL vehicle or drug dissolved in vehicle was mixed into 1 kg of powdered 5002 rodent chow (PMI Nutrition, St. Louis, MO, USA) using a stand mixer (KitchenAid, Benton Harbor, MI, USA).

### 4.2. In Vitro Experiments

Bone marrow-derived macrophages (BMDM) were isolated from femurs of adult C57BL/6 mice and differentiated using 20 ng/mL MCSF (Biolegend #576406, San Diego, CA, USA), as previously described [51]. Conditioned media was harvested from E18-14C-27 cells, derived from MMTV-Neu tumors, after 48 h of culture. BMDMs were treated using 75% conditioned media supplemented with 25% fresh media, with or without 300 nM RXR agonists for 24 h. IL-4 (10 ng/mL)(Biolegend #574304) was used as a positive control to induce a CD206+ immunosuppressive macrophage phenotype.

### 4.3. Flow Cytometry

BMDMs were harvested after 24 h treatment with conditioned media, with or without RXR agonists, filtered, and stained with fluorescent antibodies against F4/80 (APC, BM8, Biolegend) and CD206 (PE, MR6F3, Thermo Fisher Scientific). Live/dead green (Thermo Fisher Scientific) was used as a viability dye. Samples were run on BD Accuri C6 (BD Biosciences, San Jose, CA, USA.

### 4.4. In Vivo Experiments

MMTV-Neu mice [14] from our breeding colony (founders were purchased from Jackson Laboratory, Bar Harbor, ME, USA) were fed pelleted chow and palpated for tumors. Once tumors were detected, mice were switched to powder 5002 chow. Tumors were measured twice weekly with a caliper until 4 mm in diameter, at which time mice were randomized and fed control diet or 100 mg per kg per day diet of RXR agonist diet (~25 mg per kg per day body weight) for 10 days. Tumors were harvested and sections were either flash frozen for RNA-seq/qPCR or saved in neutral buffered formalin for immunohistochemistry.

### 4.5. RNA Sequencing

Frozen tumor sections (4 samples per treatment group) were weighed and homogenized. RNA was extracted using a RNeasy Mini Kit (Qiagen, Hilden, Germany), and the quality of the RNA confirmed with an Agilent Bioanalyzer (Agilent Technologies, Santa Clara, CA, USA). RNA sequencing was completed by Novogene (Sacramento, CA, USA) as described previously [52]. Raw read counts were analyzed using the DESeq2 package in R (R for Windows v. 4.1.2; R Studio v. 1.4.1717) to generate differential expression profiles, and EnrichR and Ingenuity Pathway Analysis (Qiagen) were used for enrichment analysis. Raw and processed date were deposited in the Gene Expression Omnibus and are available through GSE211290.

### 4.6. qPCR

RNA harvested from frozen tumor sections was normalized across samples using Nanodrop (Thermo Fisher Scientific), and 500 ng of RNA was used to synthesize cDNA using a High Capacity cDNA Reverse Transcription Kit (Applied Biosystems, Foster City, CA, USA). PCR was run on QuantStudio 7 Flex (Thermo Fisher Scientific) using SYBR green fluorescence. PCR data was analyzed using the delta-delta CT method using GAPDH as a housekeeping control. Error bars represent standard error of biological replicates, as indicated in figure legends. The following forward/reverse primers (Integrated DNA Technologies, Coralville, IA, USA) were used: IL-18, 5′-TCCTTGAAGTTGACGCAAGA-3′/5′-TCCAGCATCAGGACAAAGAA-3′, Col6a3, 5′ AAGGACCGTTTCCTGCTTGTT-3′/5′-GGTATGTGGGTTTCCGTTGAG-3′. Map9, 5′-GAAGAGTGCTACAGCCAACAC-3′/5′-ACAACAAGGTTTTTCCCCTTCC-3′, H2-AA, 5′-TCAGTCGCAGACGGTGTTTAT-3′/5′-GGGGGCTGGAATCTCAGGT-3′.

### 4.7. Immunohistochemistry

Formalin-fixed tissues were embedded in paraffin and sectioned by the Histology Core. Boiling citrate buffer was used for antigen retrieval, and endogenous peroxidase activity was quenched using hydrogen peroxide. Tissue sections were stained with antibodies against IL-18 (1 μg/mL, PA5-79481, Thermo Fisher Scientific), and Col6a3 (20 µg/mL, PA5-49914, Thermo Fisher Scientific), as described [34]. Sections were then labeled with biotinylated secondary antibodies (anti-rabbit, Cell Signaling Technology, Danvers, MA, USA; anti-rat, Vector Labs, Burlingame, CA, USA), as previously described. [34] A DAB substrate (Cell Signaling) was used for signal detection, as per manufacturer-provided protocols, and sections were counterstained with hematoxylin (Vector Labs). The Fiji ImageJ image processing package (version ImageJ2) was used for quantification of intensity of DAB staining by the color deconvolution method [53] and mean gray value was used to calculate optical density by the formula OD = log (max intensity/mean intensity, with a maximum intensity of 255 for 8–bit images [54].

### 4.8. KmPlot Generation

Survival curves were generated using Kaplan–Meier Plotter (https://kmplot.com/analysis/, accessed on 26 July 2022). This tool allows for correlation of gene expression to publicly available patient survival data [23]. KmPlot sources this patient data from GEO, EGA, and TCGA databases. The patient samples are split into two groups, high and low expression of the gene in question, using a robust autoselect algorithm to determine the most appropriate cutoff [24]. Breast cancer data was used, and overall or relapse free survival was compared.

### 4.9. Statistical Analysis

Results were expressed as the mean ± standard error. Data from tumor qPCR experiments were analyzed by one-tailed *t* test. *p* < 0.05 was considered statistically significant throughout all experiments. For RNAseq, differential expression analysis was performed using DESeq2. Outliers are detected by Cook’s distance and removed [55]. *p* values were adjusted to correct for multiple comparisons using the Benjamini and Hochberg method, and padj < 0.05 was considered statistically significant [56]. Data from in vitro experiments were analyzed using one-way ANOVA, and significant differences between groups were determined by the Tukey HSD multiple comparisons test.

## Data Availability

Data are contained within the article or supplementary material. The datasets generated during and analyzed during the current study are available from the corresponding author on reasonable request. For the RNA sequencing, raw and processed date were deposited in the Gene Expression Omnibus and are available through GSE211290.

## Supplementary Materials

The following supporting information can be downloaded at: https://www.mdpi.com/article/10.3390/ijms24054298/s1.
