# Supplementary material for: The Novel RXR Agonist MSU-42011 Differentially Regulates Gene Expression in Mammary Tumors of MMTV-Neu Mice
Source: Int J Mol Sci. 2023 Feb 21;24(5):4298. doi: 10.3390/ijms24054298 (PMC10001983; doi:10.3390/ijms24054298)
Supplement: Supplementary file 1 [file ijms-24-04298-s001.zip › ijms-2193225-supplementary.pptx]

## Slide 1
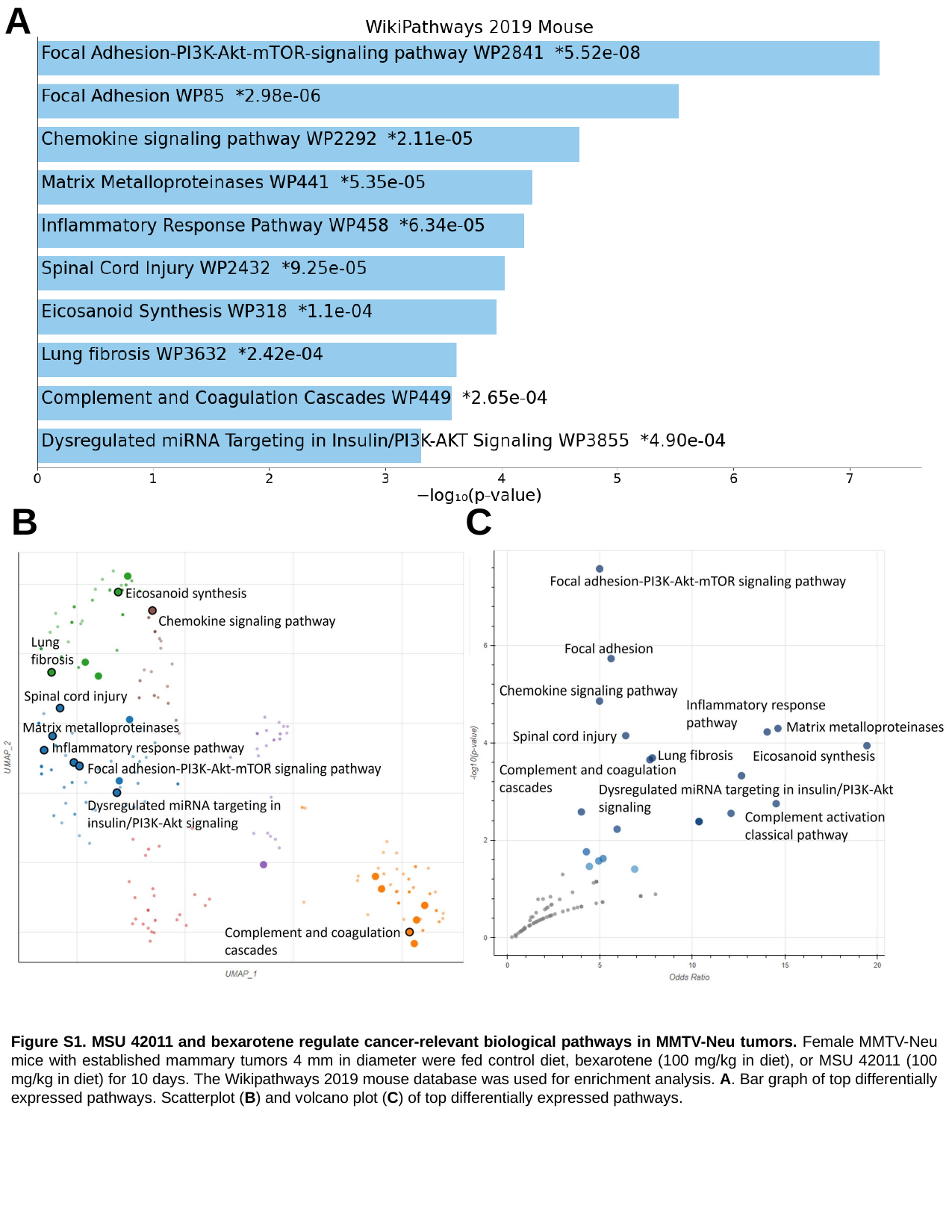

A
B
C
Figure S1. MSU 42011 and bexarotene regulate cancer-relevant biological pathways in MMTV-Neu tumors. Female MMTV-Neu mice with established mammary tumors 4 mm in diameter were fed control diet, bexarotene (100 mg/kg in diet), or MSU 42011 (100 mg/kg in diet) for 10 days. The Wikipathways 2019 mouse database was used for enrichment analysis. A. Bar graph of top differentially expressed pathways. Scatterplot (B) and volcano plot (C) of top differentially expressed pathways.

## Slide 2
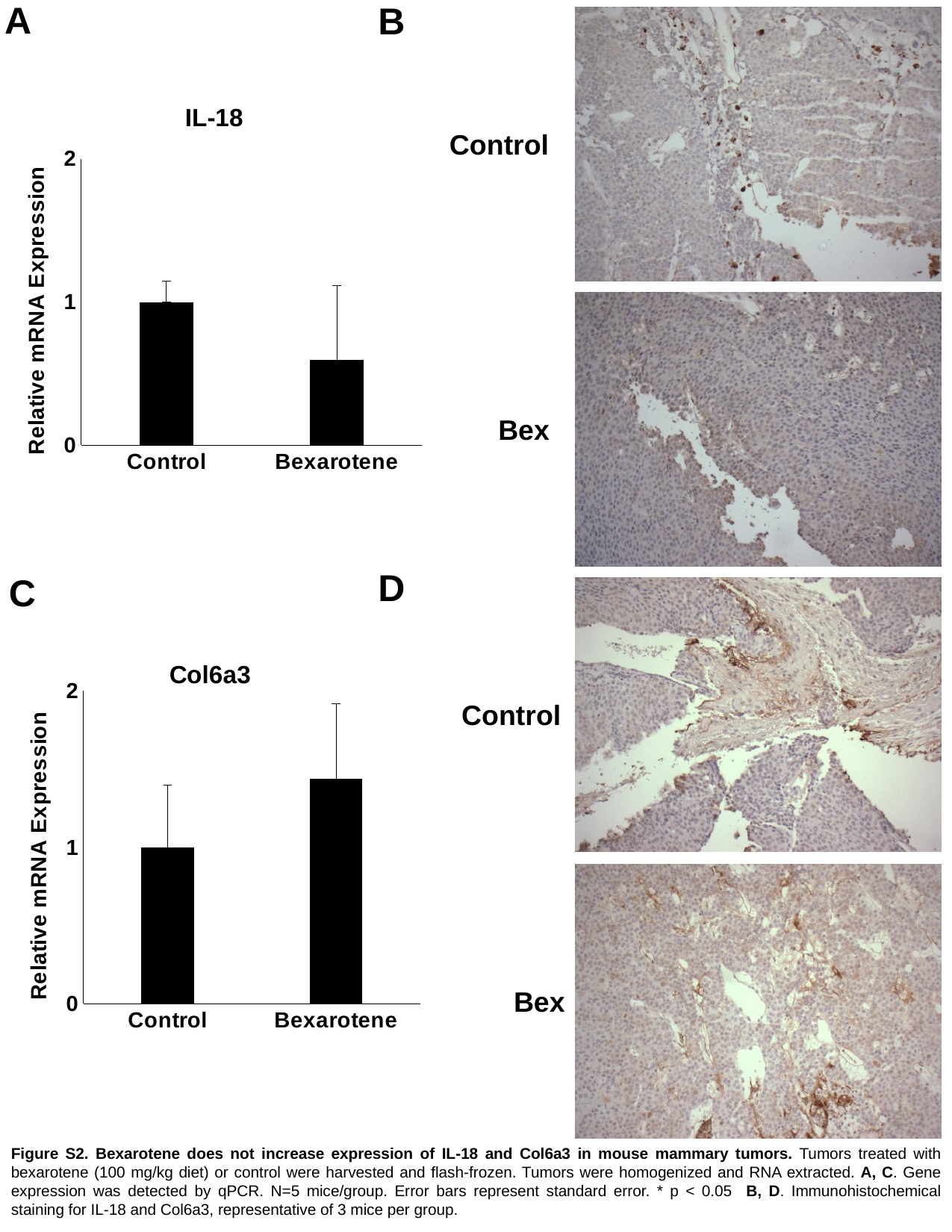

A
B
### Chart: IL-18
| Category | |
|---|---|
| Control | 1.0 |
| Bexarotene | 0.5985174246122698 |Control
Bex
D
C
### Chart: Col6a3
| Category | |
|---|---|
| Control | 1.0 |
| Bexarotene | 1.4382131193801757 |Control
Bex
Figure S2. Bexarotene does not increase expression of IL-18 and Col6a3 in mouse mammary tumors. Tumors treated with bexarotene (100 mg/kg diet) or control were harvested and flash-frozen. Tumors were homogenized and RNA extracted. A, C. Gene expression was detected by qPCR. N=5 mice/group. Error bars represent standard error. * p < 0.05 B, D. Immunohistochemical staining for IL-18 and Col6a3, representative of 3 mice per group.
